# Case Series of Incidental Findings of Low-Grade Endometrial Stromal Sarcoma on Routine Hysterectomy for Uterine Fibroids

**DOI:** 10.7759/cureus.50676

**Published:** 2023-12-17

**Authors:** Mahalakshmi Kannan, Mohanapriya Devarajan, Sudha Vasudevan

**Affiliations:** 1 Obstetrics and Gynecology, Saveetha Medical College, Saveetha Institute of Medical and Technical Sciences, Saveetha University, Chennai, IND; 2 Pathology and Laboratory Medicine, Saveetha Medical College, Saveetha Institute of Medical and Technical Sciences, Saveetha University, Chennai, IND

**Keywords:** low-grade endometrial stromal sarcoma, morcellators, cesarean hysterectomy, uterine fibroid, recurrent uterine sarcoma

## Abstract

This study is a retrospective review of data from patients who were incidentally diagnosed with endometrial stromal sarcoma after a routine hysterectomy for uterine fibroid at the Department of Gynaecologic Oncology in a tertiary care hospital. The Institutional Review Board (IRB) gave its clearance for this study before it was carried out. Informed written consent was obtained from all patients. In our case series, the three patients presented with complaints of menorrhagia, lower abdominal pain, intermenstrual bleeding, and acute retention of urine. After the patients underwent a clinical examination and radiological scan, the diagnosis of fibroids was made. They underwent a total abdominal hysterectomy with bilateral salpingo-oophorectomy. Histology revealed low-grade endometrial stromal sarcoma (LGESS). The patients were observed for adjuvant therapy. The patients had a follow-up period of 18-24 months. In LGESS, which is rare, prompt management is of utmost importance, because the tumour’s stage is the most significant predictor of the prognosis. As there are no clear indications to suggest the presence of LGESS prior to the histopathology of the specimen, it is necessary to analyze the data of patients who are diagnosed with LGESS, in order to investigate and manage the condition more appropriately. After myomectomy for a suspected leiomyoma, there is a 0.2% chance of the result showing endometrial stromal sarcoma.

## Introduction

Endometrial stromal sarcomas (ESSs) represent approximately 0.2% of all uterine tumours [1]. The WHO categorises endometrial stromal neoplasms into the following groups: endometrial stromal nodule, low-grade ESS (LGESS), high-grade ESS (HGESS), undifferentiated uterine sarcoma, and uterine tumours resembling an ovarian sex cord tumour (UTROSCT) [2].

Typically, LGESS manifests in individuals during their fourth and fifth decades of life, with a higher predominance observed in pre-menopausal women [3]. Factors such as unopposed oestrogen exposure, tamoxifen therapy, and conditions such as polycystic ovarian disease have been implicated in the pathophysiology of this condition [4]. Generally, patients present with complaints of menorrhagia, dysmenorrhoea, and lower abdominal pain, but around 25% of patients were found to be asymptomatic [5]. Approximately one-third of the patients present with extrauterine tumour extension, most commonly in the ovaries [6]. Due to the hormone sensitivity of these tumours, retaining the ovaries could result in recurrence, with rates potentially reaching up to 100% [7]. The mainstay treatment for LGESS involves surgical intervention, which typically includes a hysterectomy and bilateral salpingo-oophorectomy. In cases of recurrence or metastasis, removal of enlarged lymph nodes is indicated, where debulking surgery is considered the standard of care [8].

Additionally, adjuvant therapy can be given in the form of radiotherapy or hormonal therapy. Although LGESS has a favourable prognosis, the tumour’s stage is the most significant predictor of the prognosis. A univariate predictor of a poor outcome is when the surgical staging is greater than stage 1. The five-year survival rate for stages 1 and 2 is 90%, whereas for stages 3 and 4, it is 50%. While LGESS has low malignant potential, some cases exhibit a late recurrence post-hysterectomy [9]. Though patients present with early tumour stages, the tumour behaviour is characterised by late recurrences; as a result, long-term monitoring and follow-ups are necessary. Approximately 33% of patients with recurrence show involvement in the pelvis and abdomen, with less frequent occurrences in the vagina and lungs [2].

Studies have shown that following myomectomy for a suspected leiomyoma, there is a 0.2% chance of the findings revealing ESS [10]. In this study, we will analyze the data from three such cases.

Materials and methodology

This study is a retrospective review of data from cases with patients who were misdiagnosed with uterine fibroid and underwent hysterectomy; subsequent histopathological examination revealed that the patients had ESS at a tertiary care hospital. The Institutional Review Board (IRB) gave its clearance for this study before it was carried out. Informed written consent was obtained from all patients. Clinical and demographic details, surgical findings, histopathological findings, and survival data for all patients were collected.

## Case presentation

Case 1

A multiparous woman in her late 30s, who previously had regular menstrual cycles, presented with complaints of menometrorrhagia with intermenstrual bleeding for a period of four months. On examination, the patient had pallor; the uterus was soft and non-tender, corresponding to 14 weeks in size. Ultrasonography (USG) showed the uterus to be bulky with multiple intramural fibroids of sizes 3.6 x 4.8 cm, 4.3 x 5.1 cm, and 8.3 x 8.1 cm. The patient was diagnosed with abnormal uterine bleeding-leiomyoma (AUB-L).

Case 2

A multiparous woman in her late 40s, who previously had regular menstrual cycles, came to the out-patient department with a USG showing multiple intramural fibroids and complaints of lower abdomen pain, burning micturition, and increased frequency of micturition for two weeks. On examination, the patient had pallor, and the uterus was soft and non-tender, corresponding to a size of 12 weeks. A USG scan showed the uterus to be bulky (endometrial thickness of 8 mm) with multiple intramural fibroids of sizes 3.8 x 3 cm and 7.5 x 7.4 cm. The patient was diagnosed with AUB-L/recurrent UTI.

Case 3

**Figure 1:**
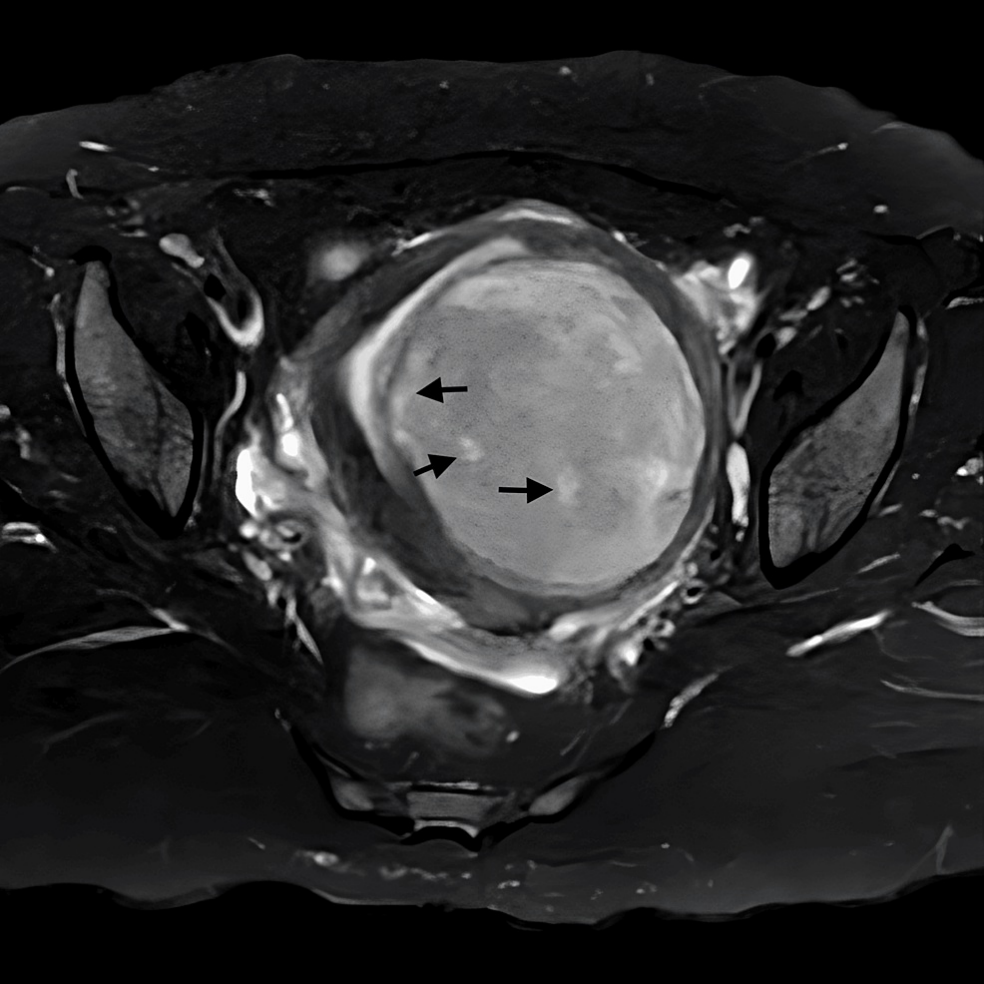
Case 3- MRI-pelvis (plain and contrast) T2 SPectral Attenuated Inversion Recovery (SPAIR) axial image showing a hyper-intense lesion with few hyper-intense foci.

**Figure 2:**
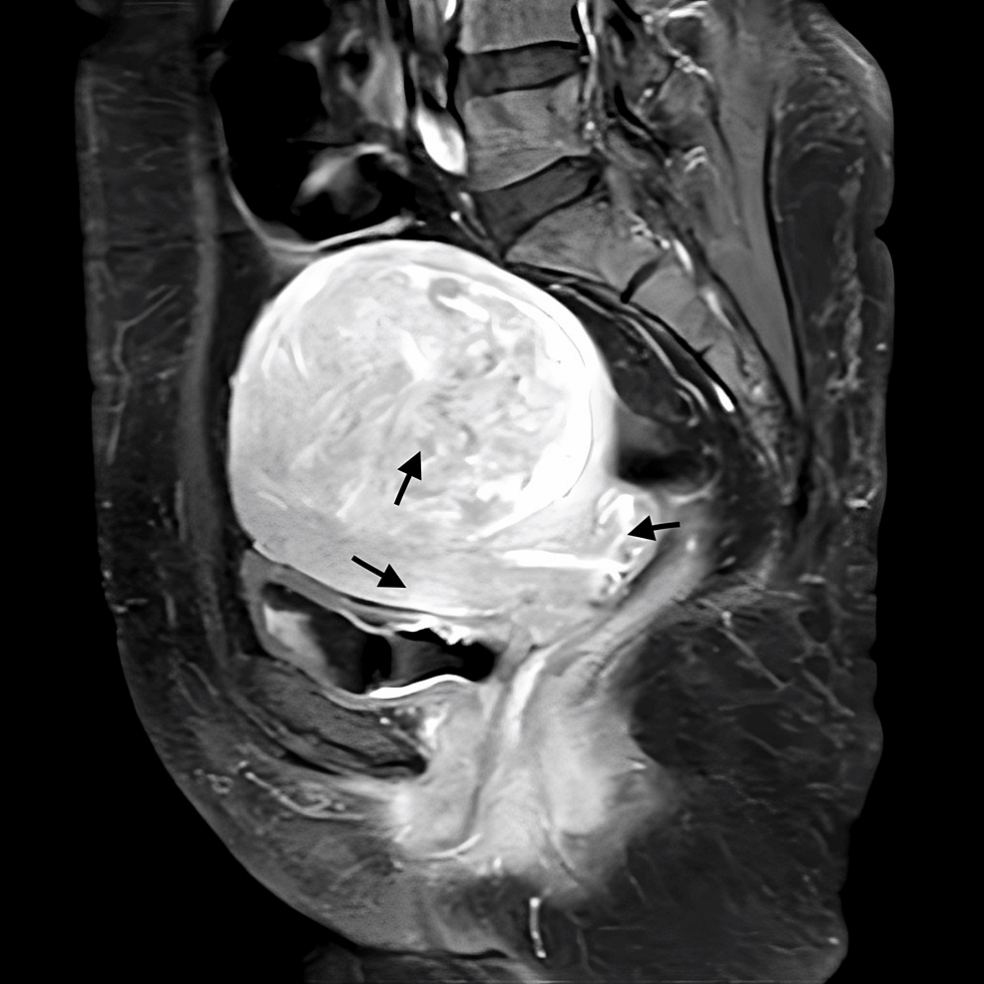
Case 3- MRI-pelvis (plain and contrast) T1 fat-saturated (T1FS) post-contrast showing a hyper-intense lesion with a whorled pattern.

A multiparous woman in her late 40s, with previous regular menstrual cycles, presented with complaints of menorrhagia, acute retention of urine in the catheter, difficulty in micturition for eight days, and fatigue on exertion. On examination, the patient had pallor, and the uterus was soft and non-tender, corresponding to a size of 12 weeks. A USG scan showed a posterior myometrial fibroid. An MRI of the abdomen and pelvis showed a bulky uterus and a submucosal/myometrial enhancing lesion along the posterior myometrium, displacing the endometrium anteriorly. The T2 SPectral Attenuated Inversion Recovery (SPAIR) axial image and T1 fat-saturated (T1FS) post-contrast image show a heterogenous hyper-intense lesion with few hyper-intense foci (Figures 1, 2, respectively). The scan was reported to show diffuse weighted Infusion (DWI) diffusion restriction with apparent diffusion coefficient (ADC) reduced diffusivity, and gradient recalled echo (GRE) with no blooming. The patient was diagnosed with AUB-L/acute retention of urine. The case scenarios for all three cases have been summarised in Table 1.

**Table 1:** Clinical presentation AUB-L: Abnormal uterine bleeding-leiomyoma; T2DM: Type 2 diabetes mellitus; SHTN: Sustained hypertension

Case number	Decade of life	Clinical features	Clinical diagnosis	Radiological diagnosis	Pre-op diagnosis	Surgery performed
1	The third decade of life	Menometrorrhagia with intermenstrual bleeding	Abnormal uterine bleeding-leiomyoma	Multiple intramural fibroids	37 year old/P2L2/AUB-L/T2DM/SHTN/severe anaemia	Total laparoscopic hysterectomy with bilateral salpingo-oophorectomy (TLH + BSO)
2	The fourth decade of life	Lower abdomen pain, burning micturition, increased frequency of micturition	Abnormal uterine bleeding-leiomyoma	Multiple intramural fibroids	47 year old/P3L3A1/AUB-L/recurrent UTI/ severe anaemia	Total abdominal hysterectomy with bilateral salpingo-oophorectomy (TAH + BSO)
3	The fourth decade of life	Metrorrhagia, acute retention of urine, difficulty in micturition, fatigue on exertion	Abnormal uterine bleeding-leiomyoma	Intramural fibroid	47 year old/P2L2/AUB-L/acute urinary retention/moderate anaemia	Total abdominal hysterectomy with right salpingo-oophorectomy and left salpingectomy (TAH+ RSO+ LS)

Gross

*Case 1* 

Grossly, the specimen was morcellated, showing a grey intramural tumour. On further evaluation, the tumour was polyp shaped, and growing into the endometrial cavity. The tumour measured about 12 x 10 x 5 cm in size with no evidence of necrosis.

Case 2

The tumour appeared grey-brown to yellow in colour. The tumour measured about 7 x 4 x 3 cm, with cystic degenerative changes seen towards the lateral side. Apart from the tumour, the specimen also showed bilateral hydrosalpinx.

Case 3

On initial evaluation of the third case, the external surface appeared congested. On the cut section, the endometrium measured 0.2 cm thick, and there were two intramural nodules measuring 8 x 6 x 4 cm (solid, lobulated, and tan brown) and 1 x 1 x 1 cm (grey-white and whorled). A cyst in the right ovary measured 1.2 cm in diameter. The tumour measured about 8 x 6 x 4 cm and showed degenerative changes. No evidence of haemorrhage or necrosis existed. Apart from the tumour, the specimen showed other features such as nabothian cysts and leiomyomas.

Microscopy

Cases 1, 2, and 3

**Figure 3:**
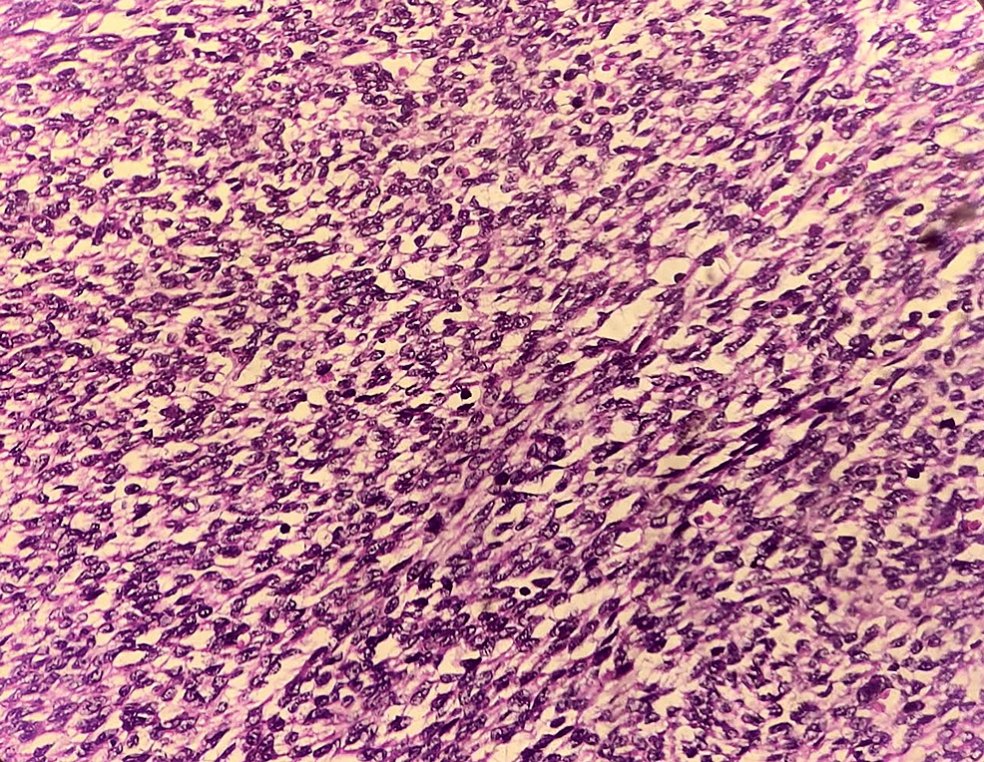
Case 1 showing endometrial stromal cells with mild atypia in microscopic examination, H&E staining (400x magnification).

**Figure 4:**
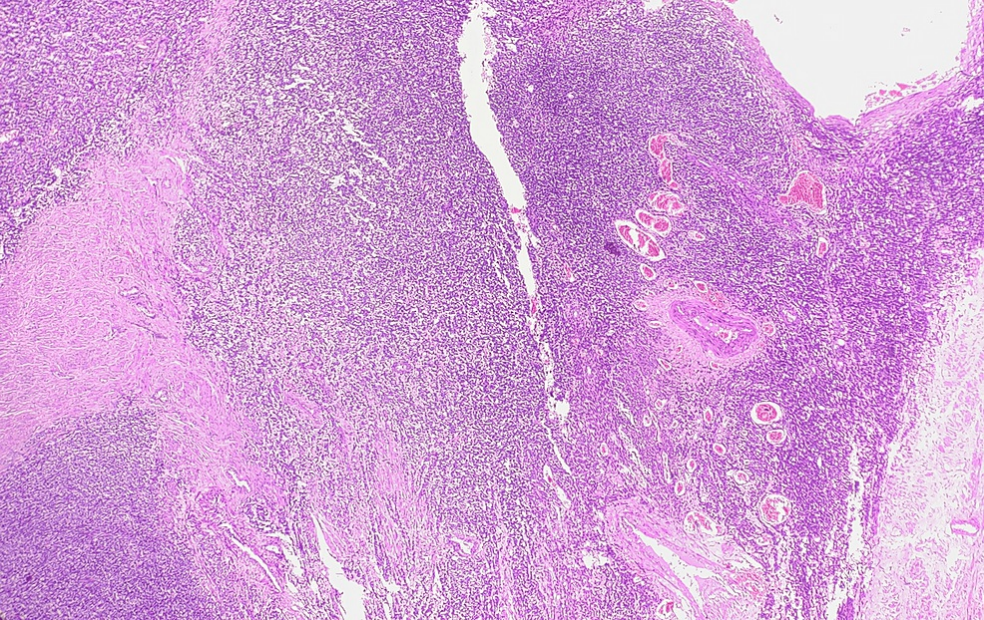
Case 2 showing well-differentiated endometrial stromal cells whirling around proliferating arterioles in microscopic examination, H&E staining (400x magnification).

**Figure 5:**
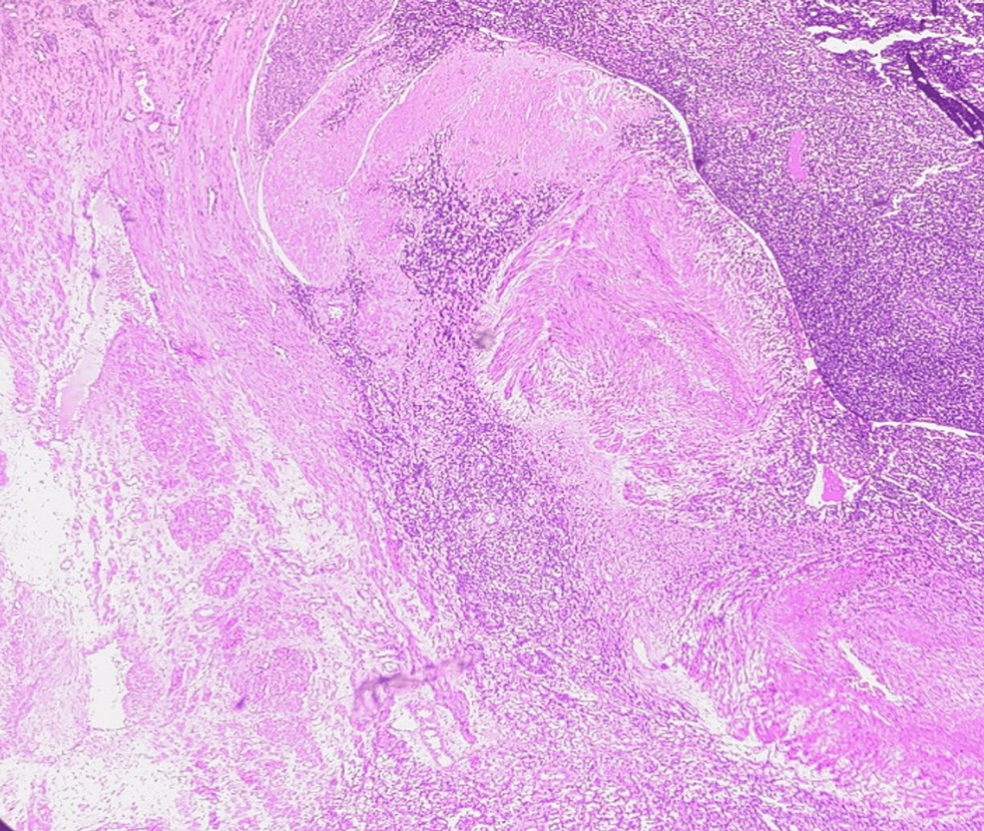
Case 3 showing endometrial stromal cells invading the myometrium in microscopic examination, H&E staining (400x magnification).

Sections showed highly cellular neoplasms constituted oval- to spindle-shaped cells and minimal cytologic atypia forming irregular cellular islands (Figure 3). The cells show a tongue-like pattern of myometrial invasion and whorls around blood vessels (Figure 4). The tumour involved more than 50% of the myometrium (Figure 5). Lympho-vascular invasion was present. No necrosis or mitotic figures were noted in all three cases. Apart from the tumour, case 1 showed features of adenomyosis. Case 3 showed endometrial hyperplasia without atypia and a leiomyoma. Immunohistochemistry was done for the third case, and the tumour was estrogen receptor (ER) and progesterone receptor (PR) positive (98%). Both gross and microscopic features have been summarised in Table 2.

**Table 2:** Histopathological features ER: Estrogen receptor; PR: Progesterone receptor; IHC: Immunohistochemistry

Case Number	Gross features	Cut section		Microscopic features			Secondary changes			Additional findings
		Size	Additional findings	Myometrial invasion	Lymphovascular invasion	IHC	Degenerative changes	Areas of necrosis	Mitosis	
1	Intramural Polypoid tumour growing into the endometrial cavity	12 x 10 x 5 cms	None	Present (>50%)	Present	Not done	None	None	None	Features of adenomyosis
2	Single intramural nodule	7 x 4 x 3 cms	Bilateral hydrosalpinx	Present	Present	Not done	Cystic degeneration over the lateral side	None	None	None
3	Two intramural nodules	8 x 6 x 4 cms	None	Absent	Absent	ER-98% positive, PR-98% positive	None	None	None	Features of endometrial hyperplasia without atypia and leiomyoma

## Discussion

In our case series, two patients presented with complaints of abnormal uterine bleeding, while only one patient presented with lower abdominal pain. All had a preoperative diagnosis of AUB-L with ultrasonographic evidence indicating intramural fibroids. Case 3 underwent pelvic MRI with contrast, confirming the presence of an intramural uterine fibroid. Cases 2 and 3 underwent open surgery, while case 1 opted for minimally invasive surgery. The frozen section of the samples were sent intra-operatively and the results were suggestive of leiomyoma in case 1 and 3. All the patients underwent total abdominal hysterectomy with bilateral salpingo-oophorectomy (TAH + BSO); in case 3, a completion surgery was performed as the left ovary was left behind in the primary surgery. 

The histopathology report of the hysterectomy specimen showed LGESS and according to the International Federation of Gynaecology and Obstetrics staging (FIGO staging), it was found to be stage 1B. These slides were revised by the pathologist for the grading to be reconfirmed, which showed LGESS (FIGO-stage 1B). As most cases are misdiagnosed as leiomyomas, it is essential for the surgeons operating on suspected leiomyomas to be aware that morcellators have to be used wisely, as they are contraindicated in ESS [11].

Following surgery, the adjunct therapy opted for all three cases was observation. The patients were monitored for a period of 18-24 months through follow-ups. At each follow-up visit, a pelvic examination and, if necessary, imaging were performed to detect tumour recurrence. Currently, all three patients are alive and disease-free. The outcome and follow-up details have been summarised in Table 3.

**Table 3:** Operative and postoperative management and prognosis of patients TLH+ BSO: Total laparoscopic hysterectomy with bilateral salpingo-oophorectomy; TAH+BSO: Total abdominal hysterectomy with bilateral salpingo-oophorectomy; TAH+ RSO+LS: Total abdominal hysterectomy with right salpingo-oophorectomy and left salpingectomy; LO: Left oophorectomy; ESS: Endometrial stromal sarcoma

Case number	Laparotomy/ laparoscopy	Primary surgery	Frozen analysis	Completion surgery	Histology	Grade	Stage	Adjuvant therapy	Follow- up	Duration of follow-up	Current status
1	Laparoscopy	TLH+ BSO	Leiomyoma	-	ESS-low grade	1	1B	Observation	Every three months	24 months	Alive and disease-free
2	Laparotomy	TAH+BSO	Not done	-	ESS-low grade	1	1B	Observation	Every three months	24 months	Alive and disease-free
3	Laparotomy	TAH+ RSO+LS	Leiomyoma	LO	ESS-low grade	1	1B	Observation	Every three months	18 months	Alive and disease-free

Due to the misdiagnosis of LGESS as leiomyoma, patients often delay treatment under the assumption of a benign condition. This delay can impact the tumour stage, subsequently influencing the patient's prognosis. Although pre-op investigations do not provide a definitive diagnosis of ESS, certain findings may raise suspicion of its presence such as fast-growing large myomas (> 6.0 cm) with an abundant blood flow signal with low resistance flow [12-14]. Findings such as DWI showing diffusion restriction, and ADC showing reduced diffusivity in MRI-pelvis with contrast may be indicative of the malignant nature of the lesion (Figures 1, 2, 3).

## Conclusions

Our study concludes the compelling need to perform USG Doppler and/or MRI-pelvis (plain and contrast) for patients with a clinical diagnosis of leiomyoma as the findings may raise suspicion of ESS. It is advised to perform a hysterectomy with bilateral oophorectomy at the earliest, preferably by a gynecologic oncologist and to use morcellators wisely in order to avoid iatrogenic dissemination. 
